# Association of Light Physical Activity Measured by Accelerometry and Incidence of Coronary Heart Disease and Cardiovascular Disease in Older Women

**DOI:** 10.1001/jamanetworkopen.2019.0419

**Published:** 2019-03-15

**Authors:** Andrea Z. LaCroix, John Bellettiere, Eileen Rillamas-Sun, Chongzhi Di, Kelly R. Evenson, Cora E. Lewis, David M. Buchner, Marcia L. Stefanick, I-Min Lee, Dori E. Rosenberg, Michael J. LaMonte

**Affiliations:** 1 Department of Family Medicine and Public Health, University of California, San Diego, La Jolla; 2 Center for Behavioral Epidemiology and Community Health (C-BEACH), Graduate School of Public Health, San Diego State University, San Diego, California; 3 Division of Public Health Sciences, Fred Hutchinson Cancer Research Center, Seattle, Washington; 4 Department of Epidemiology, Gillings School of Global Public Health, The University of North Carolina at Chapel Hill; 5 Department of Epidemiology, School of Public Health, The University of Alabama at Birmingham; 6 University of Illinois at Urbana-Champaign, Champaign; 7 Stanford Prevention Research Center, Stanford University School of Medicine, Stanford University, Stanford, California; 8 Division of Preventive Medicine, Brigham and Women’s Hospital, Harvard Medical School, Boston, Massachusetts; 9 Kaiser Permanente Washington Health Research Institute, Kaiser Permanente Washington, Seattle; 10 Department of Epidemiology and Environmental Health, School of Public Health and Health Professions, University at Buffalo, The State University of New York, Buffalo

## Abstract

**Importance:**

To our knowledge, no studies have examined light physical activity (PA) measured by accelerometry and heart disease in older women.

**Objective:**

To investigate whether higher levels of light PA were associated with reduced risks of coronary heart disease (CHD) or cardiovascular disease (CVD) in older women.

**Design, Setting, and Participants:**

Prospective cohort study of older women from baseline (March 2012 to April 2014) through February 28, 2017, for up to 4.91 years. The setting was community-dwelling participants from the Women’s Health Initiative. Participants were ambulatory women with no history of myocardial infarction or stroke.

**Exposures:**

Data from accelerometers worn for a requested 7 days were used to measure light PA.

**Main Outcomes and Measures:**

Cox proportional hazards regression models estimated hazard ratios (HRs) and 95% CIs for physician-adjudicated CHD and CVD events across light PA quartiles adjusting for possible confounders. Light PA was also analyzed as a continuous variable with and without adjustment for moderate to vigorous PA (MVPA).

**Results:**

Among 5861 women (mean [SD] age, 78.5 [6.7] years), 143 CHD events and 570 CVD events were observed. The HRs for CHD in the highest vs lowest quartiles of light PA were 0.42 (95% CI, 0.25-0.70; *P* for trend <.001) adjusted for age and race/ethnicity and 0.58 (95% CI, 0.34-0.99; *P* for trend = .004) after additional adjustment for education, current smoking, alcohol consumption, physical functioning, comorbidity, and self-rated health. Corresponding HRs for CVD in the highest vs lowest quartiles of light PA were 0.63 (95% CI, 0.49-0.81; *P* for trend <.001) and 0.78 (95% CI, 0.60-1.00; *P* for trend = .004). The HRs for a 1-hour/day increment in light PA after additional adjustment for MVPA were 0.86 (95% CI, 0.73-1.00; *P* for trend = .05) for CHD and 0.92 (95% CI, 0.85-0.99; *P* for trend = .03) for CVD.

**Conclusions and Relevance:**

The present findings support the conclusion that all movement counts for the prevention of CHD and CVD in older women. Large, pragmatic randomized trials are needed to test whether increasing light PA among older women reduces cardiovascular risk.

## Key Points

**Question:**

Is light physical activity associated with reduced risk of heart disease in older women?

**Findings:**

In this cohort study of 5861 women, the highest quartile of light physical activity was associated with a 42% reduced risk of myocardial infarction or coronary death and a 22% reduced risk of incident cardiovascular disease events compared with the lowest quartile of light physical activity. These reduced risks persisted after adjustment for sociodemographic, behavioral, and health status variables, as well as moderate to vigorous physical activity.

**Meaning:**

This study suggests that all daily life physical activity has a role in the prevention of coronary heart disease and cardiovascular disease in older women.

## Teaser

This cohort study investigates whether higher levels of light physical activity measured by accelerometry are associated with reduced risks of coronary heart disease (CHD) or cardiovascular disease (CVD) in older women.

## Introduction

Despite impressive declines in age-standardized coronary heart disease (CHD) mortality rates since the 1960s, cardiovascular disease (CVD) remains the leading cause of death in the United States and globally.^1^ More than half a million older American individuals die of CVD annually.^2^ From 1979 to 2011, declines in CHD mortality among Americans aged 65 to 84 years were faster in older men than women.^3^ While prevalence of myocardial infarction (MI) remains higher among men than women across the adult age spectrum, incidence rates of MI or fatal CHD are higher among women 85 years and older (120 per 1000) than men (85 per 1000).^4^ Yet, the prevention of CHD in older women is understudied.

Physical activity (PA) is a key candidate for reducing CHD risk in older women. The long-standing, prevailing paradigm in PA research is that moderate to vigorous PA (MVPA) for at least 150 minutes per week is needed to prevent CVD in adults. However, a meta-analysis^5^ of 9 epidemiologic studies found reduced risks of CHD associated with levels of self-reported MVPA (≥3 metabolic equivalent tasks [METs]) that were lower than the recommended guidelines. Light PA at intensity levels of 1.5 to 3.0 METs is poorly measured by self-reported questionnaires because they fail to capture light movements performed habitually throughout the day.^6,7^ Recent reports reveal that light PA measured by accelerometry is associated with reduced risks of total^8,9^ and CVD^9^ mortality, as well as favorable levels of CVD risk factors.^10^ To our knowledge, no studies have yet evaluated whether light PA is associated with reduced risks of incident CHD and CVD in adults overall or in older women specifically. The objectives of this prospective cohort study were to investigate whether device-measured light PA was associated with reduced risk of CHD or CVD in a large and diverse cohort of older women followed from the OPACH baseline (March 2012 to April 2014) and whether any associations varied by baseline levels of MVPA, estimated CVD risk, or physical functioning.

## Methods

### Study Participants

The Objectively Measured Physical Activity and Cardiovascular Health (OPACH) study is an ancillary study to the Women’s Health Initiative (WHI) that began in the early 1990s to rectify the widespread lack of data on postmenopausal women and chronic disease. Postmenopausal women aged 50 to 79 years were enrolled in the WHI clinical trials or the observational study from 40 clinical sites throughout the United States from 1993 to 1998. Between 2012 and 2014, a total of 7058 ambulatory community-dwelling women 63 years and older from the WHI were enrolled in the OPACH. Details on the WHI and OPACH have been published previously.^11,12,13^

Briefly, participants were distributed accelerometers (GT3X+; ActiGraph, LLC) to wear 24 hours per day on an elastic band over their right hip for a requested 7 days. Participants self-reported in-bed and out-of-bed times using sleep logs on days when the accelerometer was worn. Of the 6489 women who wore accelerometers, 6381 had at least 1 day with 10 or more waking hours of accelerometer wear. Women with an MI or stroke before the OPACH baseline (n = 520) were excluded, leaving 5861 women (96.1% with ≥4 days with 10 awake hours of accelerometer wear time) in the analytic study population. The protocol for this study was approved by the Fred Hutchinson Cancer Research Center Institutional Review Board, and all women provided written informed consent or telephone informed consent using an institutional review board–approved script. This report followed the Strengthening the Reporting of Observational Studies in Epidemiology (STROBE) reporting guidelines for cohort studies.

### CHD and CVD Outcome Ascertainment

From the OPACH baseline (March 2012 to April 2014) through February 28, 2017, medical updates were collected annually by mail or phone. In this report, CHD and CVD events were investigated as separate outcomes, with CHD identified as the primary end point because of its historically stronger associations with self-reported PA. Reports of incident CHD events (MI or coronary death) or incident CVD events (CHD, revascularization, carotid artery disease, hospitalized angina, congestive heart failure, stroke, or death from other CVD) were ascertained, and first events of any type were adjudicated by physician review of medical records (except angina).^14^ Defining criteria for each outcome are detailed elsewhere.^13^ There was excellent agreement among the WHI physicians on adjudication of CVD outcomes, with values ranging between 0.67 and 0.94 for κ statistics.^15^ Because older women with a prevalent CVD condition (eg, angina, heart failure, or revascularization) remain at risk for other incident manifestations of CVD, we examined incident CVD events in the same population at risk as for the CHD end point (women with no history of MI or stroke), without additional exclusion of other prevalent CVD conditions at baseline. Sensitivity analyses were conducted to determine if findings were consistent when the baseline population excluded women with the symptomatic conditions of angina and heart failure at the OPACH baseline.

### PA Measures

Accelerometer data, originally collected at 30 Hz, were aggregated to 15-second epochs using the normal frequency filter within ActiLife version 6 software (ActiGraph, LLC). Accelerometer nonwear periods were identified and removed using the Choi algorithm as previously described.^13,16^ Sleep time was removed using reported in-bed and out-of-bed times from sleep logs. Missing bed times were imputed using participant-specific mean times or, if all data were missing, the OPACH population mean (10:45 pm for in-bed time and 7:22 am for out-of-bed time).

Time spent in light PA and MVPA was computed from accelerometer data using activity intensity thresholds determined in the OPACH Calibration Study.^17^ Light PA, movements with energy expenditure measured by indirect calorimetry between 1.6 and 2.9 METs, was computed as the mean minutes per day of 15-second epochs having vector magnitude (VM) counts between 19 and 518 per day.^17^ The MVPA (METs ≥3.0) was computed as the mean minutes per day of 15-second epochs with VM counts of at least 519 per day.^17^ The PA measures were averaged over all days with awake wear time of at least 10 hours, and all such days were included in this analysis. Light PA and MVPA were adjusted for awake wear time using the residuals method to account for any systematic variations in in-bed or nonwear times.^18^

### Covariates

Potential confounders were selected based on previous literature and included age, self-identified race/ethnicity from questionnaire categories (white, black, or Hispanic/Latina), body mass index (BMI), highest education (high school or less, some college, or college graduate), current smoking (yes or no), alcohol consumption (nondrinker, <1 drink per week, ≥1 drink per week, or unknown), physical functioning (using a 10-item subscale from the RAND 36-Item Health Survey 1.0 (RAND-36),^19^ ranging from 0 [low] to 100 [high]), number of non-CVD chronic conditions (none, 1-2, or ≥3 from the sum of cancer, chronic obstructive pulmonary disease, cognitive impairment, depression, diabetes, and osteoarthritis), and systolic blood pressure, as well as self-rated health (excellent or very good; good; fair or poor). Race/ethnicity was assessed in the WHI to allow investigation of disparities. Fasting serum glucose, insulin, total cholesterol, high-density lipoprotein cholesterol (HDL-C), and high-sensitivity C-reactive protein (hsCRP) assays were conducted at the University of Minnesota Fairview Advanced Research and Diagnostic Laboratory, Minneapolis, using standardized Clinical Laboratory Improvement Act–approved methods. The Reynolds Risk Score, a strong predictor of CVD risk in the WHI cohort,^20^ was computed as previously described but without glycated hemoglobin level in women with diabetes, which was not available.

### Statistical Analysis

Participant characteristics were summarized across quartiles of light PA using means and standard deviations for continuous variables and percentages for categorical variables. *F* tests and Pearson χ^2^ tests assessed differences across quartiles for continuous and categorical variables, respectively.

Hazard ratios (HRs) for new CHD and CVD events were estimated for quartiles of light PA and MVPA (with quartile 1 as reference) using Cox proportional hazards regression. Time to event was computed as the number of days from the OPACH baseline to the date of first occurrence of a CHD or CVD event, death, or the last medical update. Regression models were progressively adjusted as follows: model 1 (n = 5861) included age and race/ethnicity; model 2 (n = 5822) added highest education, current smoking, and alcohol consumption; model 3 (n = 5750) added physical functioning, comorbidity, and self-rated health; and model 4 (n = 5861) added CVD risk factors (BMI, systolic blood pressure, hsCRP, total cholesterol, and HDL-C) thought to be in the causal pathway between PA and CVD. Biomarker data were missing from 1226 participants for whom no blood specimens were available. Therefore, models with biomarkers used data that were imputed by multivariable chained equations using 100 iterations and including CHD, CVD, both times to event, light PA, MVPA, and all covariates in the process.^21^ Model 4 results using complete case analysis are listed in eTable 1 in the Supplement. *P* values for linear trend tests were computed from Cox proportional hazards regression models that contained the continuous functional form of light PA and MVPA. Tests based on Schoenfeld residuals^22^ were used to check the proportional hazards assumptions. No violations were observed.

To examine the dose-response association of light PA (continuous variable) with CHD and CVD, restricted cubic spline functions^23^ were added to Cox proportional hazards regression model 3 with knots placed at the recommended 5th, 50th, and 95th percentiles (results were insensitive to whether 3 or 4 knots were used [eTable 2 in the Supplement]).^24^ Linearity of the dose-response association was checked using Wald tests. Dose-response trajectories were then plotted using the 10th percentile of the light PA distribution (3.3 hours per day) as the referent category.^8^ To test whether associations of light PA with CHD and CVD events were independent of MVPA, spline analyses were repeated adjusted for MVPA.

Stratified analyses were conducted to evaluate the consistency of associations across high and low levels of baseline estimated CVD risk based on the Reynolds Risk Score (median, 9.2), MVPA (median, 44.3 minutes per day), and RAND-36 physical function score (median, 75.0) defined using median splits for each variable. Hazard ratios were computed from model 3 for light PA and MVPA comparing the 75th and 25th percentiles of light PA (difference of 1.60 hours per day) and MVPA (difference of 42 minutes per day) within each strata. The statistical significance of possible effect modification was tested by adding a cross product interaction term to model 3. All variables were first mean centered to reduce multicollinearity.

All data analyses were conducted using statistical software (R, version 3.3.2; R Foundation for Statistical Computing) with the survival and rms packages. Statistical tests were all 2 sided, with the level of significance set to .05.

### Sensitivity Analyses

Models were also further adjusted for use of lipid-lowering and antihypertensive medications and for the Healthy Eating Index.^25^ Because symptoms preceding new CVD events could lead women to engage in less PA, all models were repeated after excluding CHD and CVD cases that occurred within the first 6 months of follow-up. To test whether the symptomatic conditions (angina and heart failure) were altering associations between light PA and CVD, we (1) repeated model 3 after excluding women with a history of hospitalized angina or heart failure at the OPACH baseline and (2) repeated model 3 after excluding hospitalized angina and heart failure from the CVD end point.

## Results

The mean (SD) age of 5861 OPACH participants was 78.5 (6.7) years (range, 63-99 years) (Table 1). One-third (33.5%) of the OPACH women were black, 17.6% were Hispanic, and 48.8% were of white race/ethnicity. The mean daily time spent in light PA ranged from 0.6 to 10.3 hours per day, with women in the lowest quartile having less than 3.9 hours per day and women in the highest quartile engaging in more than 5.6 hours per day. Greater proportions of younger women and black and Hispanic/Latina women were seen in the higher quartiles of light PA, but there were no differences by educational attainment. Women with more light PA had lower mean BMI, higher RAND-36 physical function scores, and lower levels of comorbidity (Table 1). As reported previously,^10^ the Reynolds Risk Score and levels of blood pressure and CVD biomarkers (glucose, insulin, and lipid levels) were more favorable among women with higher levels of light PA.

**Table 1.** Baseline Characteristics by Quartile of Time Spent in Light PA Among 5861 Women

Characteristic	No./Total No. (%)^a^				*P* Value
	Q1 (Low)	Q2	Q3	Q4 (High)	
No.	1466	1465	1465	1465	
Age, mean (SD), y	79.9 (6.7)	78.7 (6.7)	78.1 (6.6)	77.4 (6.5)	<.001
Race/ethnicity					
White	895 (61.1)	742 (50.6)	655 (44.7)	571 (39.0)	<.001
Black	399 (27.2)	490 (33.4)	524 (35.8)	553 (37.7)	
Hispanic/Latina	172 (11.7)	233 (15.9)	286 (19.5)	341 (23.3)	
BMI, mean (SD)	30.2 (6.2)	28.6 (5.5)	27.5 (5.3)	26.3 (5.1)	<.001
Highest education					
High school or less	277/1454 (19.1)	287/1455 (19.7)	287/1449 (19.8)	325/1464 (22.2)	.26
Some college	581/1454 (40.0)	580/1455 (39.9)	551/1449 (38.0)	535/1464 (36.5)	
College graduate	596/1454 (41.0)	588/1455 (40.4)	611/1449 (42.2)	604/1464 (41.3)	
Current smoker	48 (3.3)	38 (2.6)	28 (1.9)	27 (1.8)	.04
Alcohol consumption					
Nondrinker	544 (37.1)	474 (32.4)	480 (32.8)	467 (31.9)	<.001
<1 Drink/wk	471 (32.1)	476 (32.5)	465 (31.7)	433 (29.6)	
≥1 Drinks/wk	301 (20.5)	383 (26.1)	404 (27.6)	442 (30.2)	
Unknown	150 (10.2)	132 (9.0)	116 (7.9)	123 (8.4)	
RAND-36 physical function score, mean (SD)	60.5 (27.7)	68.2 (25.9)	73.7 (23.3)	76.4 (22.4)	<.001
No. of chronic conditions^b^					
None	340 (23.2)	356/1464 (24.3)	376 (25.7)	410 (28.0)	<.001
1-2	951 (64.9)	972/1464 (66.4)	963 (65.7)	945 (64.5)	
≥3	175 (11.9)	136/1464 (9.3)	126 (8.6)	110 (7.5)	
Self-rated health					
Excellent or very good	690/1461 (47.2)	727/1458 (49.9)	793/1460 (54.3)	818/1458 (56.1)	<.001
Good	596/1461 (40.8)	603/1458 (41.4)	543/1460 (37.2)	536/1458 (36.8)	
Fair or poor	175/1461 (12.0)	128/1458 (8.8)	124/1460 (8.5)	104/1458 (7.1)	
Uses antihypertensive medication	985 (67.2)	924 (63.1)	908 (62.0)	873 (59.6)	<.001
Uses antilipidemic medication	620 (42.3)	641 (43.8)	549 (37.5)	513 (35.0)	<.001
Light PA, mean (SD), min/d	196.0 (32.2)	262.2 (14.2)	309.6 (14.0)	379.6 (38.8)	<.001
Reynolds Risk Score, mean (SD)	16.2 (13.2)	12.7 (10.3)	11.5 (9.5)	9.6 (8.2)	<.001
MVPA, mean (SD), min/d	34.2 (25.8)	47.2 (29.8)	56.5 (33.7)	66.2 (35.7)	<.001
Blood pressure, mean (SD), mm Hg					
Systolic	127.6 (15.2)	126.0 (13.9)	124.8 (13.8)	124.1 (13.7)	<.001
Diastolic	73.5 (9.4)	73.0 (8.5)	72.5 (8.4)	71.6 (8.4)	<.001
hsCRP, mean (SD), mg/L^c^	0.8 (1.1)	0.7 (1.0)	0.6 (1.0)	0.4 (1.0)	<.001
Cholesterol, mean (SD), mg/dL					
Total	195.4 (40.0)	198.1 (39.6)	199.9 (39.4)	202.5 (38.1)	<.001
HDL	56.6 (13.8)	59.8 (14.1)	62.1 (15.3)	64.1 (15.2)	<.001

Abbreviations: BMI, body mass index (calculated as weight in kilograms divided by height in meters squared); HDL, high-density lipoprotein; hsCRP, high-sensitivity C-reactive protein; MVPA, moderate to vigorous PA; PA, physical activity; Q, quartile.

SI conversion factors: To convert cholesterol level to millimoles per liter, multiply by 0.0259; to convert hsCRP level to nanomoles per liter, multiply by 9.524.

^a^ Adjusted for awake wear time using the residuals method. Quartile cut points are 36 to 236 min/d for Q1, 237 to 285 min/d for Q2, 286 to 333 min/d for Q3, and 334 to 617 min/d for Q4. For some variables in the table, totals are less than the column headings because of missing data.

^b^ Cancer, chronic obstructive pulmonary disease, cognitive impairment, depression, diabetes, and osteoarthritis.

^c^ Natural log transformed.

A total of 143 incident cases of CHD and 570 incident cases of CVD occurred during 20 718 person-years of follow-up (mean, 3.53 years; range, 0.01-4.91 years) (Table 2). The CHD HR comparing the highest vs lowest quartiles adjusted for age and race/ethnicity (model 1) was 0.42 (95% CI, 0.25-0.70; *P* for trend <.001). The HR after model 3 adjustments was 0.58 (95% CI, 0.34-0.99; *P* for trend = .004). The HR after further model 4 adjustment for CVD risk factors, including BMI, systolic blood pressure, hsCRP, total cholesterol, and HDL-C, was 0.68 (95% CI, 0.39-1.18; *P* for trend = .03; 42% lower risk of CHD).

**Table 2.** Associations of Incident CHD and CVD With Light Physical Activity and MVPA in the Objectively Measured Physical Activity and Cardiovascular Health (OPACH) Cohort (2012-2017)

Outcome and Model^a^	HR (95% CI)^b^				*P* Value for Trend^c^
	Q1 (Low)	Q2	Q3	Q4 (High)	
**Light PA**					
Incident CHD events (crude incidence rate per 1000 person-years)	59 (11.8)	36 (7.0)	28 (5.4)	20 (3.8)	NA
Model 1	1 [Reference]	0.67 (0.44-1.01)	0.55 (0.35-0.87)	0.42 (0.25-0.70)	<.001
Model 2	1 [Reference]	0.71 (0.47-1.08)	0.60 (0.38-0.96)	0.46 (0.28-0.78)	<.001
Model 3	1 [Reference]	0.79 (0.51-1.20)	0.72 (0.45-1.15)	0.58 (0.34-0.99)	.004
Model 4	1 [Reference]	0.82 (0.54-1.26)	0.79 (0.49-1.27)	0.68 (0.39-1.18)	.03
Incident CVD events (crude incidence rate per 1000 person-years)	183 (37.9)	161 (32.3)	124 (24.3)	102 (19.7)	NA
Model 1	1 [Reference]	0.93 (0.75-1.15)	0.73 (0.58-0.92)	0.63 (0.49-0.81)	<.001
Model 2	1 [Reference]	0.96 (0.78-1.19)	0.77 (0.61-0.97)	0.66 (0.52-0.85)	<.001
Model 3	1 [Reference]	1.02 (0.82-1.27)	0.88 (0.69-1.11)	0.78 (0.60-1.00)	.004
Model 4	1 [Reference]	1.05 (0.84-1.30)	0.90 (0.71-1.14)	0.82 (0.63-1.07)	.02
**MVPA**					
Incident CHD events (crude incidence rate per 1000 person-years)	77 (15.6)	25 (4.9)	24 (4.6)	17 (3.2)	NA
Model 1	1 [Reference]	0.38 (0.24-0.61)	0.42 (0.26-0.68)	0.34 (0.19-0.59)	<.001
Model 2	1 [Reference]	0.40 (0.25-0.63)	0.44 (0.27-0.71)	0.38 (0.22-0.67)	<.001
Model 3	1 [Reference]	0.46 (0.29-0.72)	0.55 (0.34-0.90)	0.54 (0.30-0.96)	.001
Model 4	1 [Reference]	0.45 (0.28-0.72)	0.58 (0.36-0.95)	0.58 (0.32-1.04)	.003
Incident CVD events (crude incidence rate per 1000 person-years)	229 (48.7)	143 (28.7)	106 (20.6)	92 (17.5)	NA
Model 1	1 [Reference]	0.68 (0.55-0.84)	0.54 (0.42-0.68)	0.50 (0.39-0.65)	<.001
Model 2	1 [Reference]	0.69 (0.56-0.86)	0.55 (0.44-0.71)	0.53 (0.41-0.69)	<.001
Model 3	1 [Reference]	0.77 (0.62-0.96)	0.65 (0.51-0.84)	0.69 (0.53-0.91)	.009
Model 4	1 [Reference]	0.75 (0.61-0.93)	0.66 (0.52-0.84)	0.71 (0.54-0.93)	.02

Abbreviations: CHD, coronary heart disease; CVD, cardiovascular disease; HR, hazard ratio; MVPA, moderate to vigorous physical activity; NA, not applicable; PA, physical activity; Q, quartile.

^a^ Data used for model 4 were imputed because biomarker data were missing from 1226 women. Results from complete case analysis are listed in eTable 1 in the Supplement. Regression models were progressively adjusted as follows: model 1 (n = 5861) included age and race/ethnicity; model 2 (n = 5822) added highest education, current smoking, and alcohol consumption; model 3 (n = 5750) added physical functioning, comorbidity, and self-rated health; and model 4 (n = 5861) added CVD risk factors (body mass index, systolic blood pressure, high-sensitivity C-reactive protein, total cholesterol, and high-density lipoprotein cholesterol) thought to be in the causal pathway between PA and CVD.

^b^ Adjusted for awake wear time using the residuals method. Quartile cut points for light PA are 36 to 236 min/d for Q1, 237 to 285 min/d for Q2, 286 to 333 min/d for Q3, and 334 to 617 min/d for Q4. Quartile cut points for MVPA less than 26 min/d for Q1, 27 to 44 min/d for Q2, 45 to 68 min/d for Q3, and 69 to 350 min/d for Q4.

^c^ *P* values from Cox proportional hazards regression models that include light PA as a continuous variable.

Associations between light PA and incident CVD events followed a similar pattern (Table 2). The CVD HRs comparing the highest vs lowest quartiles were 0.63 (95% CI, 0.49-0.81; *P* for trend <.001) after minimal adjustment (model 1), 0.78 (95% CI, 0.60-1.00; *P* for trend = .004) after adjustment for confounders (model 3), and 0.82 (95% CI, 0.63-1.07; *P* for trend = .02; 18% lower risk of CVD) after inclusion of CVD risk factors likely to be in the causal pathway (model 4).

For MVPA, HRs indicated statistically significant risk reductions for both CHD and CVD beginning at quartile 2, which corresponds to 27 minutes or more of MVPA daily, agreeing well with PA guidelines (Table 2). The HRs comparing the women in the highest vs lowest MVPA quartiles after adjusting for model 3 confounders were 0.54 (95% CI, 0.30-0.96; *P* for trend = .001; 46% lower risk of CHD) for CHD and 0.69 (95% CI, 0.53-0.91; *P* for trend = .009; 31% lower risk of CVD) for CVD.

**Figure 1.**
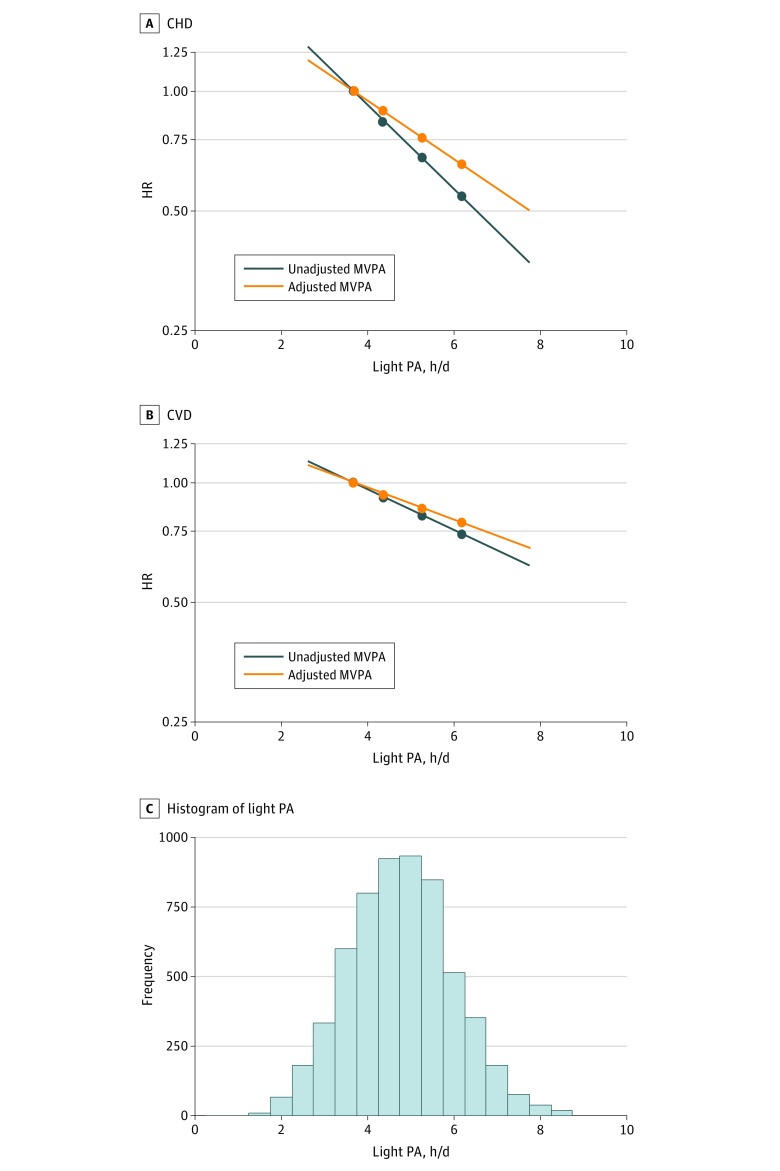
Continuous Dose-Response Association of Light Physical Activity (PA) With Coronary Heart Disease (CHD) and Cardiovascular Disease (CVD) Events A, Association with incident CHD events. B, Association with incident CVD events. C, Distribution of daily light PA for the Objectively Measured Physical Activity and Cardiovascular Health (OPACH) cohort. All associations were estimated using multivariable linear Cox proportional hazards regression models adjusted for age, race/ethnicity, highest education, current smoking, alcohol consumption, physical functioning, comorbidity, and self-rated health (blue lines). Orange lines show results after additional adjustment for moderate to vigorous PA (MVPA). The reference category was set to the 10th percentile of light PA (3.3 hours per day). Respective hazard ratios (HRs) and 95% CIs for 4, 5, and 6 hours per day of light PA (compared with the reference) were for CHD: not adjusted for MVPA 0.84 (0.75-0.95), 0.68 (0.52-0.88), 0.54 (0.36-0.82); adjusted for MVPA 0.89 (0.79-1.00), 0.76 (0.58-1.00), 0.65 (0.42-1.01). For CVD: not adjusted for MVPA 0.92 (0.87-0.97), 0.83 (0.73-0.94), 0.74 (0.61-0.91); adjusted for MVPA 0.94 (0.88-1.00), 0.86 (0.75-0.99), 0.79 (0.64-0.98). Results were trimmed at the 1st and 99th percentiles.

Analyzing light PA as a continuous variable, the risk for incident CHD and CVD events decreased in a linear dose-dependent manner over increasing light PA levels (eTable 2 in the Supplement). Hazard ratios adjusting for potential confounders (model 3) for each 1-hour increment in light PA were 0.80 (95% CI, 0.69-0.93; *P* for trend = .004) for CHD (Figure 1A) and 0.90 (95% CI, 0.83-0.97; *P* for trend = .004) for CVD (Figure 1B). Adjustment for MVPA (model 4) slightly attenuated associations, with HRs for 1 hour of light PA changing to 0.86 (95% CI, 0.73-1.00; *P* for trend = .05) for incident CHD events and to 0.92 (95% CI, 0.85-0.99; *P* for trend = .03) for incident CVD events.

**Figure 2.**
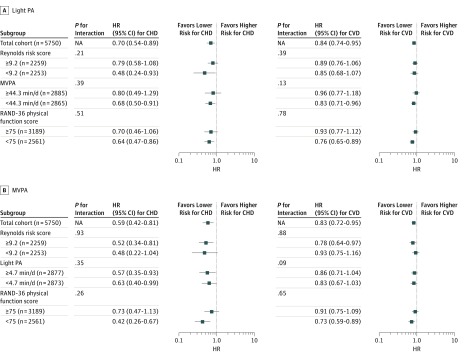
Associations of Physical Activity (PA) With Coronary Heart Disease (CHD) and Cardiovascular Disease (CVD) Events, by Selected Participant Characteristics A, Associations comparing the 75th vs 25th percentiles of light PA (difference of 1.6 hours per day) with incident CHD and CVD events. B, Associations comparing the 75th vs 25th quartiles of moderate to vigorous PA (MVPA) (difference of 42 minutes per day) with incident CHD and CVD events. Hazard ratios (HR) were adjusted for age, race/ethnicity, highest education, current smoking, alcohol consumption, physical functioning, comorbidity, and self-rated health (where appropriate). Reynolds Risk Score, MVPA, physical functioning, and light PA were split at the median. Hazard ratios below 1 indicate favorable associations (ie, lower risk), whereas those above 1 indicate harmful associations (ie, higher risk). NA indicates not applicable; error bars, 95% CIs. The n values for subanalyses stratified by Reynolds Risk Score do not sum to 5750 because of missing biomarker data.

As shown in Figure 2 comparing women in the 75th vs 25th percentiles of light PA, reduced risks of incident CHD events were observed across high and low levels of Reynolds Risk Score and RAND-36 physical function score. Hazard ratios for light PA appeared stronger among women with low MVPA, but the interaction was not statistically significant. Similar results were observed in stratified analyses for incident CVD events. For MVPA, HRs were somewhat stronger than for light PA for CHD overall (HR for the 75th vs 25th percentiles, 0.59; 95% CI, 0.42-0.81) and in some strata. No statistically significant interactions were observed between MVPA and any of the stratifying factors (Figure 2). No statistically significant interactions were observed between light PA and race/ethnicity for either the CHD or CVD outcomes.

Sensitivity analyses indicated that results were unchanged when CHD or CVD events that occurred during the first 6 months of follow-up were excluded or when additional adjustments were made for use of lipid-lowering medication, antihypertensive medication, and the Healthy Eating Index. Results were also unchanged when women with angina and heart failure at the OPACH baseline were excluded from the analytic sample and when angina and heart failure were excluded from the CVD end point.

## Discussion

In this prospective cohort study of older women, light PA measured by accelerometry was associated with a dose-responsive, independent reduced risk of incident CHD and CVD events. The highest quartile of light PA was associated with a 42% reduced risk of MI or coronary death and a 22% reduced risk of incident CVD events compared with the lowest quartile of light PA. These reduced risks persisted after multivariable adjustment that included physical functioning and other measures of health status, even though some covariates may themselves be altered by PA and thus dilute the associations. The reduced risks of CHD and CVD were also statistically significant after simultaneous adjustment for MVPA. In this study, intensity of PA was classified using a triaxial accelerometer VM count cut point specifically calibrated in a clinic-based study^17^ for older women. To our knowledge, this is the first study to investigate accelerometer-measured light PA in relation to incident CHD, including nonfatal and fatal events in older women.

The majority of active time in older adults is spent in light PA, which contributes about equally to daily PA energy expenditure as MVPA in older people.^26^ Yet, little is known about the cardiovascular consequences of light PA. Previous studies^5^ on the dose response between PA and CHD risk have focused on amounts of self-reported MVPA, not on the entire range of PA intensity that could be associated with benefit. A major barrier has been that self-reported questionnaires measuring leisure-time PA do not adequately capture light PA that is acquired throughout the day in activities of daily living. In the OPACH cohort, there was essentially no correlation between light PA measured by the WHI physical activity questionnaire^27,28^ and by accelerometry (*r* = 0.03).^29^ In a recent analysis of National Health and Nutrition Examination Survey data,^8^ US adults 40 years and older who spent 5 or more hours per day in accelerometer-measured light PA had a 23% lower risk of mortality compared with those who spent less than 3 hours per day in light PA (HR, 0.77; 95% CI, 0.60-1.00). However, other reports found no association of accelerometer-measured light PA with total or CVD mortality.^30,31^ By contrast, the OPACH women in the highest vs lowest tertiles of low light PA (19-225 VM counts per 15 seconds) had a 36% reduction in risk of CVD mortality (95% CI, 0.41-0.99) after adjustments similar to those of the present study, and women in the highest tertile of high light PA (226-518 VM counts per 15 seconds) had a 70% reduced risk of CVD mortality (95% CI, 0.17-0.51).^9^ The inconsistencies among these previous results may be due to differences in study populations, length of follow-up, cut points used to classify PA intensity, approaches to adjustment, and statistical power, as well as whether or not early events and deaths were excluded to account for reverse causality. The strong, independent associations of light PA with reduced risks of incident CHD and CVD in the present study add notably to this growing evidence base because both fatal and nonfatal incident CVD events were studied.

Associations of light PA with incident CHD are biologically plausible. In the OPACH cohort, women who engaged in greater light PA had more favorable baseline levels of HDL-C and low-density lipoprotein cholesterol, triglycerides, glucose, CRP, BMI, and Reynolds Risk Score.^20^ Adjustment for CVD risk factors attenuated associations between light PA and first CHD or CVD events, supporting the possibility that light PA alters CHD risk partially, but not completely, through its association with these risk factors. Accelerometer-measured light PA has also been associated with lower levels of subclinical atherosclerosis, including carotid femoral pulse wave velocity and carotid intima media thickness in older men.^32^

The present findings are consistent with a large body of evidence showing that self-reported MVPA reduces risk of CHD and CVD in the United States and worldwide.^5,33^ In the OPACH cohort, women in the highest quartile of MVPA had a 46% reduced risk of incident CHD and a 31% reduced risk of CVD events compared with their less active peers in the lowest quartile (Table 2). For light PA, the same comparisons yielded risk reductions of 42% and 22% for CHD and CVD, respectively. The magnitude of these associations for light PA and their consistency across strata of CVD risk, physical functioning, and MVPA suggest that light PA could have much to offer older women in the prevention of CVD whether or not they can or choose to engage in MVPA.

### Strengths and Limitations

This prospective study had numerous strengths, including the large, diverse cohort of women. Substantial representation of women older than 80 years provides evidence in an understudied but increasingly numerous segment of the US population. Inclusion of fatal and nonfatal physician-adjudicated CHD and CVD end points is a major strength. Use of accelerometers with calibrated age-appropriate cut points for distinguishing light PA from MVPA is a major and unique strength of this study. Resting metabolic rate declines with age,^34^ and the energy costs of activity increase with age.^35,36^ The OPACH Calibration Study^17^ showed that the typically used National Health and Nutrition Examination Survey cut points^37^ result in underestimation of both MVPA and light PA. We were not able to examine relative intensity, which requires individual calibration with maximal exercise testing. For some women, a MET value of 1.5 to 3.0 could fall within the moderate range of PA intensity. The study had up to 5 years of follow-up and was conducted only among older women. However, our results appear generalizable to men given a recent study^38^ of 1181 older British men that reported an HR for light PA of 0.74 (95% CI, 0.41-1.34) for incident CVD events, which is remarkably similar to the HR of 0.78 (95% CI, 0.60-1.00) in the present study. Longer-term prospective studies with inclusion of both sexes are needed to increase the strength of the evidence base on light PA in relation to CVD prevention.

## Conclusions

In 2016, an estimated 25% of US women 75 years and older met federal PA guidelines for aerobic activity,^39^ which require 75 minutes of vigorous activity or 150 minutes of moderate activity per day. These guidelines may have discouraged PA when perceived to be unattainable by large segments of the population. The present findings support the newly released 2018 Physical Activity Guidelines Advisory Committee Scientific Report, which states that “[f]or individuals who perform no or little moderate-to-vigorous physical activity, replacing sedentary behavior with light-intensity physical activity reduces the risk of all-cause mortality, cardiovascular disease incidence and mortality”^40^^(pA-4)^ and suggests that “all movement counts” when it comes to CHD and CVD prevention in older women. Large randomized trials, such as the ongoing Women’s Health Initiative Strong and Healthy Study (WHISH^41^), are needed to conclusively determine whether pragmatic interventions can increase light PA among older women and whether doing so reduces the occurrence of CVD. Given the low risks of light PA and the abundance of light movements that are part of everyday life, even in the absence of definitive trial data, it may be prudent to encourage older women to increase light PA to improve their CVD health and reduce the occurrence of CVD events.

## Notes

Supplement. **eTable 1.** Hazard Ratios and 95% CIs From Multivariable Cox PH Models With and Without Multiple Imputation **eTable 2.** P Values From Restricted Cubic Spline Cox Regression Models and From Linear Cox Regression Models
