# Typographical Error in Conclusions

**DOI:** 10.1001/jamanetworkopen.2019.4476

**Published:** 2019-05-10

In the Original Investigation titled “Association of Light Physical Activity Measured by Accelerometry and Incidence of Coronary Heart Disease and Cardiovascular Disease in Older Women,”^1^ a typographical error in the Conclusions of the article body was present on initial publication. The first sentence of the Conclusions should read, “…150 minutes of moderate activity per week.” This article has been corrected.^1^
