# Supplementary material for: Association of Light Physical Activity Measured by Accelerometry and Incidence of Coronary Heart Disease and Cardiovascular Disease in Older Women
Source: JAMA Netw Open. 2019 Mar 15;2(3):e190419. doi: 10.1001/jamanetworkopen.2019.0419 (PMC6484645; doi:10.1001/jamanetworkopen.2019.0419)
Supplement: Supplement. — eTable 1. Hazard Ratios and 95% CIs From Multivariable Cox PH Models With and Without Multiple Imputation eTable 2. P Values From Restricted Cubic Spline Cox Regression Models and From Linear Cox Regression Models [file jamanetwopen-2-e190419-s001.pdf]

Supplementary Online Content

LaCroix AZ, Bellettiere J, Rillamas-Sun E, et al; Women's Health Initiative (WHI). Association of light physical activity measured by accelerometry and incidence of coronary heart disease and cardiovascular disease in older women. *JAMA Netw Open*. 2019;2(3):e190419. doi:10.1001/jamanetworkopen.2019.0419

eTable 1. Hazard Ratios and 95% CIs From Multivariable Cox PH Models With and Without Multiple Imputation

eTable 2. P Values From Restricted Cubic Spline Cox Regression Models and From Linear Cox Regression Models

This supplementary material has been provided by the authors to give readers additional information about their work.

© 2019 LaCroix AZ et al. *JAMA Network Open*.

eTable 1. Hazard ratios and 95% CIs from multivariable Cox PH models with and without multiple imputation

	Light Physical Activity Quartiles[a,b]				P-Trend[c]
	**1 (low)**	**2**	**3**	**4 (high)**	
**Coronary heart disease (CHD)**					
Model 4[d] (complete case analysis)[e]	1 (ref)	0.73 (0.45-1.18)	0.75 (0.44-1.29)	0.71 (0.39-1.29)	0.05
Model 4[d] (multiple imputation analysis)[f]	1 (ref)	0.80 (0.52-1.22)	0.74 (0.46-1.18)	0.61 (0.36-1.04)	**0.007**
**Cardiovascular disease (CVD)**					
Model 4[d] (complete case analysis)[e]	1 (ref)	1.03 (0.81-1.32)	0.94 (0.72-1.24)	0.87 (0.65-1.17)	0.10
Model 4[d] (multiple imputation analysis)[f]	1 (ref)	1.05 (0.84-1.30)	0.90 (0.71-1.14)	0.82 (0.63-1.07)	**0.02**
	**Moderate to Vigorous Physical Activity Quartiles[a,b]**				
	**1 (low)**	**2**	**3**	**4 (high)**	
**Coronary heart disease (CHD)**					
Model 4[d] (complete case analysis)[e]	1 (ref)	0.44 (0.26-0.74)	0.49 (0.28-0.86)	0.53 (0.28-1.02)	**0.001**
Model 4[d] (multiple imputation analysis)[f]	1 (ref)	0.45 (0.28-0.72)	0.58 (0.36-0.95)	0.58 (0.32-1.04)	**0.003**
**Cardiovascular disease (CVD)**					
Model 4[d] (complete case analysis)[e]	1 (ref)	0.79 (0.62-1.01)	0.64 (0.49-0.85)	0.73 (0.54-0.99)	**0.04**
Model 4[d] (multiple imputation analysis)[f]	1 (ref)	0.75 (0.61-0.93)	0.66 (0.52-0.84)	0.71 (0.54-0.93)	**0.02**

[a] Quartile (Q) cutpoints (min.): Light physical activity Q1 = 36-236 min, Q2 = 237-285 min, Q3 = 286-333 min, Q4 = 334-617 min Moderate to vigorous physical activity Q1=-18-26, Q2=27-44, Q3=45-68, Q4=69-350.

[b] Adjusted for awake wear time using the residuals method.

[c] P-values from Cox multivariable linear regression models including physical activity in models in continuous form.

[d] Data are hazard ratio (95% confidence interval). Model 4 is adjusted for age, race-ethnicity, education, current smoking, alcohol consumption, comorbidity, physical functioning, self-rated health, BMI, systolic blood pressure, total cholesterol, HDL-cholesterol, and log(CRP).

[e] n = 4351.

[f] n = 5861.

© 2019 LaCroix AZ et al. *JAMA Network Open*.

eTable 2. P-values from restricted cubic spline cox regression models and from linear cox regression models

	P-values[a] from restricted cubic spline models		P-values[a] from linear models
	3 knots	4 knots	
**Light PA[b] adjusted for covariates[c] (not MVPA)**			
**CVD**			
Overall[d]	**0.02**	**0.03**	**0.004**
Nonlinear	0.86	0.75	n/a
**CHD**			
Overall[d]	**0.01**	**0.02**	**0.004**
Nonlinear	0.59	0.46	n/a
**Light PA[b] adjusted for covariates[c] and MVPA**			
**CVD**			
Overall[d]	0.10	0.17	**0.03**
Nonlinear	0.89	0.75	n/a
**CHD**			
Overall[d]	0.13	0.15	0.05
Nonlinear	0.64	0.50	n/a

Abbreviations: PA = Physical activity; MVPA = Moderate to vigorous PA; CHD = Coronary heart disease; CVD = Cardiovascular disease

[a] P-values are from Wald tests.

[b] Light PA and MVPA were adjusted for awake wear time using the residuals method.

[c] All models were adjusted for age, race-ethnicity, education, current smoking, alcohol consumption, comorbidity, physical functioning, self-rated health.

[d] For restricted cubic spline models, the overall model contains light PA in its linear and nonlinear functional form. For the linear models, the overall model contains light PA in its linear functional form.
